# Interoceptive dysfunction and its neural correlates in schizophrenia: protocol for a cross-sectional multimodal MRI study

**DOI:** 10.3389/fpsyt.2026.1766350

**Published:** 2026-04-17

**Authors:** Peipei Luan, Mengmeng Fan, Ronghong Gao, Anpei Wei, Ting Liu, Xiaoyan He, Zhaoguo Liu

**Affiliations:** 1 Department of Psychiatry, The Affiliated Mental Health Center of Jiangnan University, Wuxi, Jiangsu, China; 2 School of Humanities and Management Science, Wannan Medical College,Wuhu, Anhui, China; 3 General Surgery Department, Wuxi Huishan District People’s Hospital, Wuxi, Jiangsu, China

**Keywords:** diffusion tensor imaging, heartbeat counting task, interoception, multimodal neuroimaging, resting-state functional MRI, schizophrenia, self-disturbance

## Abstract

**Background:**

Interoception—the perception and integration of internal bodily signals—is fundamental to emotion regulation, bodily self-awareness, and predictive coding. Emerging evidence suggests that interoceptive disturbances may contribute to core psychopathological features of schizophrenia. Our research group recently conducted a systematic review and meta-analysis demonstrating significant impairments in interoceptive accuracy and sensitivity among individuals with schizophrenia. However, the neural mechanisms underlying these deficits remain unclear.

**Methods:**

This cross-sectional protocol will recruit 30 individuals with schizophrenia and 30 age- and sex-matched healthy controls. Participants will complete (1) behavioral interoceptive assessment using the heartbeat counting task; (2) subjective interoceptive questionnaires, including the Multidimensional Assessment of Interoceptive Awareness (MAIA) and the Body Perception Questionnaire (BPQ); (3) clinical symptom ratings (PANSS, HAM-A, HAM-D); and (4) cognitive testing (TMT, animal fluency, DSST). All participants will undergo multimodal MRI scanning, including structural T1-weighted imaging, resting-state fMRI, and diffusion tensor imaging. Neuroimaging data will be preprocessed and analyzed using DPABISurf, SPM12, and GRETNA. Expected Results: We anticipate that individuals with schizophrenia will show reduced interoceptive accuracy, altered subjective interoceptive awareness, and abnormal intrinsic neural activity and connectivity within interoception-related circuits, including the anterior insula, anterior cingulate cortex, amygdala, and thalamus. Structural abnormalities within thalamo-cortical pathways are also expected. Interoceptive deficits are hypothesized to correlate with symptom severity and cognitive performance.

**Conclusions:**

This study will provide an integrated characterization of interoceptive dysfunction and its neural correlates in schizophrenia. Findings may advance understanding of bodily self-disturbance and emotional dysregulation and support the development of future interoception-focused therapeutic approaches.

**Clinical trial registration:**

https://www.chictr.org.cn/, identifier ChiCTR2500110551.

## 1 Introduction

Schizophrenia spectrum disorders are heterogeneous psychiatric conditions characterized by positive symptoms (e.g., hallucinations and delusions), disorganized thinking or behavior, and negative symptoms such as diminished emotional expression and avolition, accompanied by varying degrees of cognitive and functional impairment. Epidemiological studies estimate a lifetime prevalence of schizophrenia spectrum disorders of approximately 0.3–0.7%, although estimates vary across populations and geographic regions. Individuals with schizophrenia spectrum disorders have a reduced life expectancy compared with the general population, reflecting a combination of physical health comorbidities, lifestyle factors, and healthcare disparities (1, 2). These conditions are associated with challenges in emotional regulation, cognitive functioning, and social participation, particularly given their typical onset in early adulthood and recurrent symptom fluctuations, highlighting the importance of improving mechanistic understanding and clinical care (3). Although considerable progress has been made in elucidating the neurobiological underpinnings of schizophrenia, its pathophysiology remains only partially understood, and clinical diagnosis continues to rely predominantly on subjective symptom assessment rather than objective biomarkers (4).

Interoception—the sensing, interpretation, and integration of internal bodily signals—has emerged as a central construct in contemporary psychiatry and neuroscience. Interoceptive processing involves hierarchical neural systems that encode and integrate visceral and physiological signals to generate dynamic representations of the internal bodily state. Primary interoceptive signals are conveyed via spinal and vagal afferent pathways to the posterior insular cortex, where they are represented in a modality-specific manner. These signals are subsequently integrated in higher-order regions, including the anterior insula and anterior cingulate cortex, which support emotional awareness, self-referential processing, and adaptive regulation of behavior. Through this hierarchical integration across distributed neural circuits, interoception contributes to emotional regulation, self-awareness, decision-making, and adaptive functioning (5, 6). Recent theoretical frameworks, including predictive coding models, propose that interoception arises from hierarchical inference processes integrating sensory signals with prior expectations about bodily states (7, 8).

Interoceptive functions can be examined through validated behavioral paradigms, such as the heartbeat counting task (HCT) (9), as well as through subjective self-report measures, including the Multidimensional Assessment of Interoceptive Awareness (MAIA) and the Body Perception Questionnaire (BPQ) (10, 11). Although the heartbeat counting task (HCT) is one of the most widely used paradigms for assessing interoceptive accuracy, its construct validity has been increasingly debated in recent literature. Several studies have suggested that HCT performance may be influenced not only by interoceptive ability itself but also by factors such as beliefs about heart rate and time estimation ability (12–14). Despite these limitations, the HCT remains widely used in psychiatric and schizophrenia research, allowing comparability with previous studies. To address potential confounding effects related to temporal perception, the present study additionally includes a time estimation task and will account for time estimation performance in the statistical analysis.

Growing research suggests that altered interoceptive processing may contribute to the disturbances in bodily self-awareness and affective dysregulation observed in schizophrenia (15, 16). Our recent systematic review and meta-analysis (17) provided comprehensive quantitative evidence demonstrating significant and consistent impairments in both interoceptive accuracy and interoceptive sensitivity in individuals with schizophrenia. These findings establish interoceptive dysfunction as a robust and measurable feature of schizophrenia and highlight its relevance to broader disturbances in bodily self-awareness, emotional processing, and self–other differentiation (18, 19). However, despite strong behavioral evidence, the neural mechanisms underlying interoceptive impairments in schizophrenia remain insufficiently understood. In particular, few studies have directly examined the relationships between behavioral interoceptive performance and alterations in functional and structural brain networks implicated in interoceptive processing (20–22).

Taken together, current evidence highlights interoception as a promising but understudied dimension of psychopathology in schizophrenia. Although behavioral and subjective interoceptive impairments have been documented, the neurobiological mechanisms that give rise to these abnormalities remain largely unknown. Although interoceptive processing has been linked to a distributed set of brain regions, the specific functional and structural substrates of interoceptive impairments in schizophrenia remain insufficiently characterized. Functional MRI studies have consistently implicated the insular cortex as a core hub, with the posterior insula supporting primary interoceptive representations and the anterior insula integrating interoceptive signals with salience processing and affective evaluation. Additional regions frequently involved include the anterior cingulate cortex (ACC), somatosensory cortex, thalamus, and prefrontal areas, reflecting integration of visceral signals with attention, emotion, and self-related processing. In parallel, DTI research suggests that interoceptive functions rely on the integrity of white-matter pathways connecting these regions, including thalamocortical projections and fronto-limbic association tracts (e.g., cingulum bundle and uncinate fasciculus), which may support communication between insula/ACC hubs and distributed cortical networks. However, it remains unclear how alterations in intrinsic brain activity, functional connectivity, and white-matter microstructure across these interoception-related systems jointly contribute to behavioral interoceptive deficits in schizophrenia (23, 24). A multimodal approach integrating behavioral assessment, self-report measures, and neuroimaging is therefore essential to advance mechanistic understanding in this area.

To address this gap, the present study proposes a comprehensive investigation of interoceptive dysfunction in schizophrenia using validated behavioral paradigms and a multimodal MRI framework. By examining intrinsic neural activity (rs-fMRI), connectivity patterns, and structural integrity (Diffusion Tensor Imaging, DTI), alongside standardized symptom ratings and cognitive assessments, this study aims to identify neural correlates of interoceptive dysfunction and clarify their associations with clinical features. Findings from this research may provide mechanistic insight into bodily self-disturbance in schizophrenia and contribute to the development of objective biomarkers and future interoception-focused intervention strategies.

## 2 Methods

**Figure 1:**
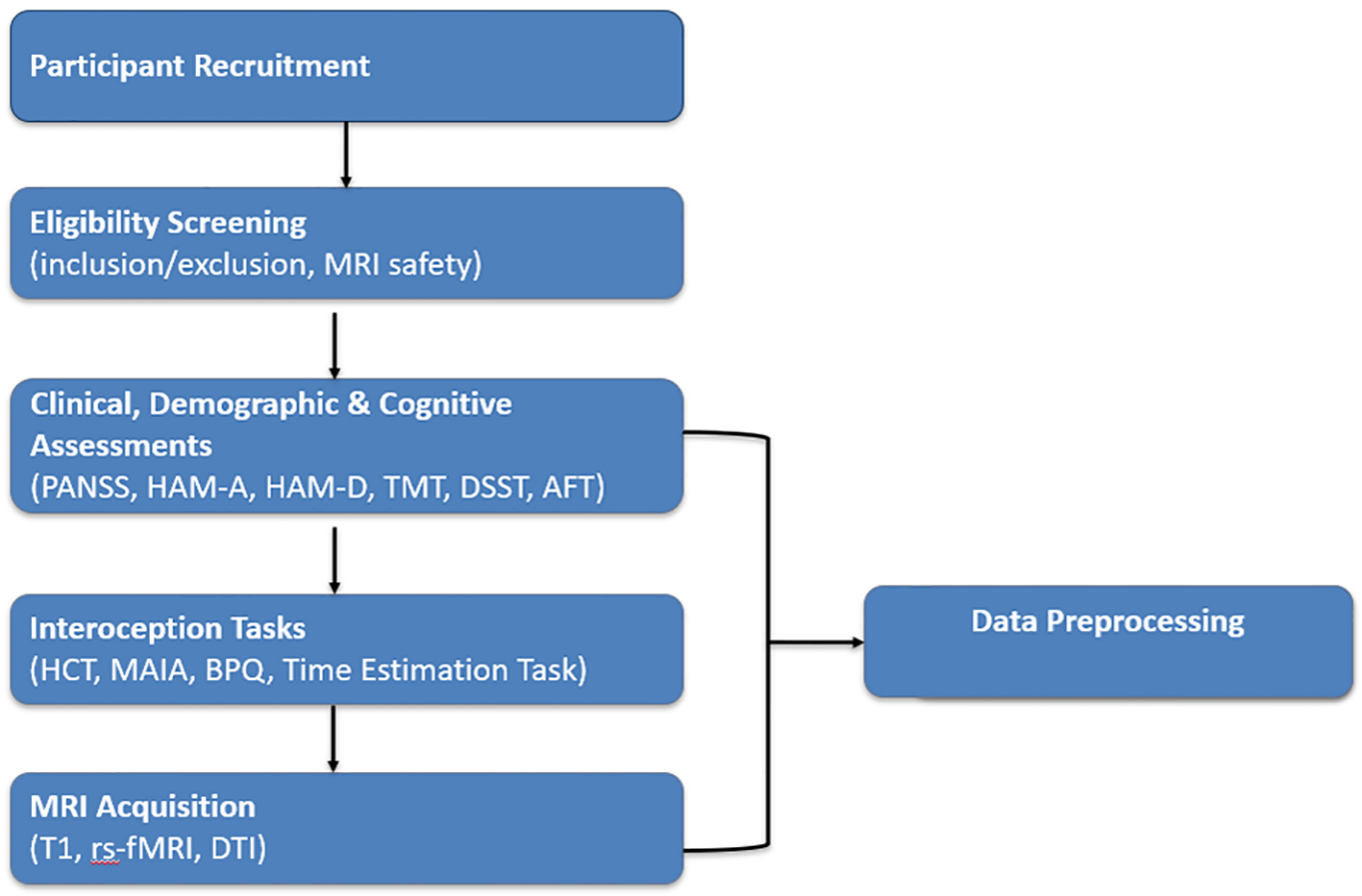
Study workflow. Overview of participant recruitment, eligibility screening, behavioral and clinical assessments(PANSS, HAM-A, HAM-D, TMT, DSST, AFT), interoception tasks (HCT, MAIA, BPQ, Time Estimation Task), MRI acquisition, and data preprocessing.

The overall workflow of participant recruitment, assessment procedures, and MRI acquisition is illustrated in **Figure 1**.

### 2.1 Objectives

#### 2.1.1 Primary objectives

1. To compare interoceptive accuracy between individuals with schizophrenia and matched healthy controls using the heartbeat counting task (HCT).
2. To examine group differences in subjective interoceptive awareness using validated self-report instruments, including the MAIA and the BPQ.

#### 2.1.2 Secondary objectives

1. To investigate associations between interoceptive impairments (behavioral and subjective) and clinical symptom severity, including positive, negative, and general psychopathology (Positive and Negative Syndrome Scale, PANSS), as well as comorbid anxiety and depression (Hamilton Anxiety Scale, HAM-A, Hamilton Depression Scale HAM-D).
2. To evaluate relationships between interoceptive accuracy and cognitive performance, including processing speed (Digit Symbol Substitution Test, DSST), executive functioning (Trail Making Test, TMT), and verbal fluency (Animal Fluency Test).

#### 2.1.3 Exploratory objectives

1. To characterize abnormalities in intrinsic neural activity within interoception-related regions (e.g., anterior insula, anterior cingulate cortex, amygdala, thalamus) using resting-state fMRI metrics such as amplitude of low-frequency fluctuations (ALFF), regional homogeneity (ReHo), and functional connectivity.
2. To assess structural alterations in white-matter pathways implicated in interoceptive processing using DTI indices, including fractional anisotropy (FA), mean diffusivity (MD), radial diffusivity (RD), and axial diffusivity (AD).
3. To examine brain–behavior associations by linking neural abnormalities with interoceptive accuracy, subjective interoceptive awareness, clinical symptoms, and cognitive performance.
4. To evaluate performance on a time estimation task and explore its associations with interoceptive accuracy, subjective interoceptive awareness, clinical symptoms, and cognitive functioning.
5. To identify potential multimodal neural markers of interoceptive dysfunction in schizophrenia.

### 2.2 Study design

This study adopts a cross-sectional, observational design comparing individuals with schizophrenia and matched healthy controls. All participants will undergo standardized assessment procedures, including behavioral interoceptive tasks, subjective questionnaires, clinical symptom ratings, cognitive tests, and multimodal MRI scanning. The study procedures follow a fixed sequence to ensure consistency across participants. Data collection is conducted at Wuxi Mental Health Center. The study protocol has received ethical approval from the institutional review board and has been registered in the Chinese Clinical Trial Registry (ChiCTR2500110551).

### 2.3 Participants

Participants will include 30 individuals with schizophrenia and 30 age- and sex-matched healthy controls. The planned sample size was informed by effect sizes reported in our recent meta-analysis examining interoceptive accuracy in schizophrenia (17). That study reported a moderate effect size for group differences in interoceptive accuracy between individuals with schizophrenia and healthy controls. Based on this effect size, a sample of approximately 30 participants per group provides adequate statistical power (approximately 80%) to detect group differences at an alpha level of 0.05. In addition, sample sizes of approximately 25–35 participants per group are commonly reported in multimodal neuroimaging studies investigating schizophrenia. Therefore, the present sample size was considered appropriate for the behavioral objectives of this study.

Individuals with schizophrenia will be recruited from inpatient and outpatient units at Wuxi Mental Health Center and diagnosed by two senior psychiatrists according to DSM-5 criteria. Healthy controls will be recruited from the local community through advertisements and word-of-mouth and screened To ensure the absence of psychiatric, neurological, or major medical conditions. All participants will be right-handed, aged 18–65 years, and capable of providing written informed consent.

#### 2.3.1 Inclusion criteria (schizophrenia group)

1. Diagnosis of schizophrenia confirmed by two attending or senior psychiatrists according to DSM-5 criteria.
2. Age between 18 and 65 years.
3. Right-handedness (Right-handedness was required to minimize variability related to hemispheric lateralization of brain structure and function. Hemispheric dominance has been shown to influence functional connectivity patterns, regional activation, and white-matter organization in MRI studies. Restricting the sample to right-handed individuals helps reduce inter-individual variability and improves the interpretability of group-level analyses of functional and structural neuroimaging measures, particularly in regions implicated in interoceptive processing, such as the insula and anterior cingulate cortex).
4. Stable antipsychotic medication regimen for at least two weeks or medication-naïve (status will be recorded for analysis).
5. Ability to understand study procedures and provide informed consent.

#### 2.3.2 Exclusion criteria (both groups)

1. History of neurological disorders, severe head injury, or seizure disorders.
2. Current substance or alcohol use disorder (past 6 months).
3. Major medical or endocrine disorders that may influence interoceptive processing (e.g., severe cardiovascular disease, diabetes with autonomic neuropathy).
4. Pregnancy or breastfeeding.
5. Contraindications to MRI (e.g., implanted metallic devices, severe claustrophobia).
6. Intellectual disability or severe cognitive impairment was assessed using standardized cognitive screening measures, including the Montreal Cognitive Assessment (MoCA), in conjunction with clinical evaluation and functional history.

#### 2.3.3 Additional exclusion criteria for healthy controls

1. Lifetime history of any psychiatric disorder (assessed using a structured clinical interview).
2. First-degree relatives with a psychotic disorder.

Written informed consent will be obtained from all participants prior to enrollment. The study protocol was approved by the Ethics Committee of Wuxi Mental Health Center (Approval No: WXMHCIRB2025LLky094) and registered in the Chinese Clinical Trial Registry (ChiCTR2500110551).

### 2.4 Procedures

All participants will complete a standardized assessment session consisting of demographic collection, clinical evaluations, interoceptive assessments, cognitive testing, and MRI scanning. All procedures will follow a fixed, predetermined order to minimize variability across participants. Assessments will be conducted by trained research personnel who are blinded to the study hypotheses.

#### 2.4.1 Enrollment and screening

Participants with schizophrenia will be approached in clinical units, whereas healthy controls will be recruited through community advertisements and word-of-mouth. Eligibility screening will include demographic information, psychiatric and medical history, and MRI safety evaluation. Written informed consent will be obtained prior to participation.

#### 2.4.2 Clinical and demographic assessments

Participants will undergo standardized clinical evaluations, including: PANSS, HAM-A, HAM-D, demographic information (age, sex, education and so on) and illness duration and medication status (patients only). All clinical ratings will be performed by two trained psychiatrists to ensure inter-rater reliability.

#### 2.4.3 Cognitive assessments

Validated neuropsychological tests will be administered to assess cognitive performance: Trail Making Test (TMT): processing speed and executive function, Animal Fluency Test (AFT): verbal fluency and Digit Symbol Substitution Test (DSST): processing speed and attention. All cognitive assessments will be conducted in a quiet room to minimize distractions.

#### 2.4.4 Interoceptive assessments

##### 2.4.4.1 Heartbeat counting task

Interoceptive accuracy was assessed using the heartbeat counting task (HCT), a widely used behavioral paradigm for evaluating cardiac interoceptive perception (9, 25). Participants will complete three trials of varying durations (25 s, 35 s, 45 s). Instructions including Sit comfortably and remain still. Focus attention internally on heartbeat sensations. Avoid checking the pulse manually or using counting strategies. Report the number of perceived heartbeats after each interval. A 30-second practice trial will precede the formal task. Interoceptive accuracy will be calculated using the standard HCT formula.

##### 2.4.4.2 Subjective interoceptive measures

Participants will complete two validated self-report questionnaires: MAIA and BPQ. Questionnaires will be administered using paper forms.

##### 2.4.4.3 Time estimation task

Temporal perception was assessed using a time estimation task, a widely used paradigm in interval timing research and frequently applied in studies of predictive processing and schizophrenia (26, 27). Participants will also complete a time estimation task to assess temporal perception; a cognitive domain theoretically related to interoception and predictive processing. Time estimation is closely linked to interoceptive processing, internal predictive models, and disturbances in self-monitoring—domains known to be altered in schizophrenia (26).Participants will estimate the duration of predefined intervals (19 s, 37 s, 49 s) without counting, external cues, or motor strategies. They will verbally report their estimate after each interval.

Three practice trials will be administered before the formal assessment. Absolute estimation error (|estimated – actual|) and relative error [(estimated – actual)/actual] will be recorded for later analyses. For specific details, please refer to the **Supplementary Materials Text S1**.

#### 2.4.5 MRI scanning procedures

MRI scanning will be conducted at Wuxi Ninth People’s Hospital, where MRI facilities are available for the present study.

Preparation.

1. Participants will remove all metal objects and complete MRI safety screening.
2. Earplugs and padding will be provided to reduce noise and minimize head motion.
3. Participants will be instructed to remain still, keep their eyes closed, and stay awake during resting-state scans.

Sequence Order.

A fixed acquisition order will be used:

1. Structural T1-weighted MRI (MPRAGE).
2. Resting-state fMRI.
3. Diffusion tensor imaging (DTI).

Total scanning duration will be approximately 30 minutes. MRI technicians will monitor data quality throughout acquisition. Parameter settings are detailed in **Supplementary Material Table 1**.

#### 2.4.6 Data storage and quality control

1. All data will be coded with unique participant identifiers.
2. Raw and preprocessed MRI data will be stored on secure, password-protected servers.
3. Quality control will include checks for excessive head motion (>2.5 mm translation or >2.5°rotation).
4. Datasets failing quality control will be excluded according to predefined criteria.

A detailed schedule of screening, baseline evaluation, behavioral testing, and MRI acquisition is presented in **Table 1**.

**Table 1:** Study schedule of participant assessment.

Assessment domain	Screening	Enrollment/baseline	Assessment session	MRI session
Eligibility review	X			
Informed consent		X		
Demographics		X		
Medical/psychiatric history	X	X		
MRI safety screening	X			X
Clinical assessments			X	
PANSS			X	
HAM-A			X	
HAM-D			X	
Cognitive assessments			X	
TMT			X	
DSST			X	
AFT			X	
Interoceptive tasks			X	
Heartbeat Counting Task (HCT)			X	
MAIA			X	
BPQ (124-item)			X	
Time Estimation Task			X	
MRI scanning				X
T1-weighted structural scan				X
Resting-state fMRI				X
DTI				X
Data Quality Control				X

“X” indicates that the specified assessment is conducted at the corresponding study phase.

### 2.5 Outcomes

#### 2.5.1 Primary outcome

Interoceptive accuracy, assessed using the heartbeat counting task (HCT).

Interoceptive accuracy will be quantified using the standard HCT formula across the three intervals(25 s, 35 s, 45 s).

#### 2.5.2 Secondary outcomes

1. Subjective interoceptive awareness, evaluated using MAIA and BPQ.
2. Objective–subjective interoception relationships, including correlations between HCT accuracy and MAIA subscales and BPQ total/subscale scores.
3. Clinical symptom severity, including PANSS, anxiety symptoms (HAM-A) and depressive symptoms (HAM-D).
4. Cognitive performance, assessed by: Trail Making Test (TMT), Digit Symbol Substitution Test (DSST) and Animal Fluency Test (AFT).
5. Associations among interoceptive measures (behavioral and subjective), clinical symptoms, and cognitive performance, including correlations between HCT/MAIA/BPQ and PANSS, HAM-A, HAM-D, TMT, DSST, and AFT scores.

#### 2.5.3 Exploratory outcomes

1. Intrinsic neural activity, derived from resting-state fMRI metrics: Amplitude of Low-Frequency Fluctuations (ALFF), Regional Homogeneity (ReHo), Seed-based and ROI-to-ROI functional connectivity within interoception-related regions and networks, including: anterior insula, anterior cingulate cortex, amygdala, thalamo–cortical pathways and salience network nodes.
2. Structural connectivity, assessed using DTI (DTI) indices: fractional anisotropy (FA), mean diffusivity (MD), radial diffusivity (RD), axial diffusivity (AD). Analyses will focus on interoception-related white-matter tracts, including fronto–limbic and thalamo–cortical pathways.
3. Brain–behavior associations, examining relationships among: functional connectivity and DTI indices, interoceptive accuracy (HCT), subjective interoceptive awareness (MAIA, BPQ), clinical symptom severity and cognitive performance.
4. Multimodal neural markers, identifying integrated structural–functional features associated with interoceptive dysfunction in schizophrenia.
5. Time estimation performance, measured as absolute and relative error during the time estimation task, and its associations with interoceptive measures, clinical symptoms, and cognitive performance.

For specific details, please refer to **Supplementary Material Table 2**.

### 2.6 Data analysis

#### 2.6.1 Behavioral and questionnaire data

1. All behavioral, clinical, and questionnaire analyses will be performed using SPSS 26.0 and R 4.3.0.
2. Group comparisons: Continuous variables (HCT accuracy, time estimation performance, MAIA, BPQ, TMT, AFT, DSST) will be compared using independent-samples t-tests or Mann–Whitney U tests for non-normal distributions. Categorical variables will be analyzed using χ² tests.
  All analyses will control for age, sex, years of education, and medication dosage (chlorpromazine equivalents). In analyses involving HCT accuracy, time estimation performance will additionally be included as a covariate to control for potential confounding effects related to temporal estimation ability. Additional sensitivity analyses will examine whether group differences in HCT accuracy remain significant after controlling for time estimation performance.
3. Correlation analyses: Within each group, associations between interoceptive accuracy, subjective measures (MAIA/BPQ), PANSS scores, and cognitive performance will be examined using Pearson or Spearman correlations with FDR correction.

#### 2.6.2 MRI data preprocessing

1. MRI preprocessing will be conducted using DPABISurf 1.8 (based on fMRIPrep), SPM12, GRETNA for network analysis, and the FMRIB Software Library (FSL) for diffusion tensor imaging analysis.
2. The rs-fMRI preprocessing steps include: (1) slice timing correction; (2) motion correction; (3) co-registration to structural T1 images; (4) nuisance regression (motion parameters, white matter, and CSF signals); (5) spatial normalization to MNI space; (6) band-pass filtering (0.01–0.08 Hz); and (7) spatial smoothing with a 6 mm FWHM Gaussian kernel.

Participants with excessive head motion (> 2.5 mm translation or > 2.5° rotation) will be excluded from further analysis. In addition, framewise displacement (FD) will be calculated as a quality-control metric to assess motion during scanning, and scans with excessive motion (e.g., mean FD > 0.5 mm) will be carefully inspected.

#### 2.6.3 rs-fMRI metrics

1. ALFF and ReHo.
  Group comparisons will be performed using voxel-wise two-sample t-tests with Gaussian Random Field (GRF) correction (voxel-level p < 0.001, cluster-level p < 0.05).
2. Functional Connectivity (FC).
  Seed regions include the bilateral anterior insula, dorsal anterior cingulate cortex (dACC), amygdala, thalamus, and nodes of the salience network.

Analytic steps include: (1) extraction of seed time series; (2) computation of Pearson correlation matrices; (3) Fisher’s Z-transformation; and (4) group comparison of FC maps using GRF correction.

#### 2.6.4 DTI analysis

DTI data will be processed using FSL. The following diffusion indices will be extracted: fractional anisotropy (FA), mean diffusivity (MD), radial diffusivity (RD), and axial diffusivity (AD).

Tract-Based Spatial Statistics (TBSS) will be used to create a white-matter skeleton, followed by group comparisons with FDR correction.

Although the present analyses focus on conventional DTI metrics (FA, MD, RD, and AD), the multi-shell acquisition protocol also provides the possibility of applying advanced diffusion models, such as Neurite Orientation Dispersion and Density Imaging (NODDI) or constrained spherical deconvolution (CSD), in future analyses to further characterize white matter microstructure. Advanced diffusion modeling approaches, such as NODDI, have increasingly been applied to characterize microstructural abnormalities in schizophrenia (28, 29).

#### 2.6.5 Multimodal brain–behavior integration

Integrated analyses will evaluate relationships among neural indices (ALFF, ReHo, FC, FA, MD, RD, AD), interoceptive accuracy (HCT), subjective interoceptive awareness (MAIA, BPQ), clinical symptoms (PANSS, HAM-A, HAM-D), and cognitive performance.

Statistical methods will include multiple linear regression and partial correlations (controlling for covariates), with Bonferroni or FDR correction applied for multiple comparisons. The MRI preprocessing pipeline is illustrated in **Supplementary Figure 1**.

#### 2.6.6 Handling of missing data

Missing data will be addressed using pairwise deletion for correlation analyses and multiple imputation when missingness exceeds 5%.

A study-specific Case Report Form (CRF) was developed to ensure standardized acquisition of demographic, clinical, interoceptive, cognitive, and MRI-related data. The complete CRF is included as a **Supplementary File**. The full multimodal analysis pipeline is provided in **Supplementary Figure 2**.

## 3 Discussion

This protocol outlines a comprehensive and multimodal investigation into interoceptive dysfunction in schizophrenia, integrating behavioral performance, subjective awareness, clinical characteristics, cognitive assessments, and multimodal neuroimaging. Building on our previous meta-analysis demonstrating significant impairments in interoceptive accuracy and sensitivity in individuals with schizophrenia, this study is positioned to extend prior work by examining the neurobiological substrates underlying these deficits. The proposed design allows systematic characterization of intrinsic neural activity, functional connectivity, and white-matter microstructure within interoception-related circuits, including the anterior insula, dorsal anterior cingulate cortex, amygdala, and thalamo-cortical pathways.

By incorporating both objective behavioral measures (heartbeat counting task) and subjective assessments (MAIA and BPQ), the study adopts a multidimensional approach aligned with contemporary theoretical models of interoceptive processing. The integration of clinical and cognitive assessments further enables examination of how interoceptive dysfunction relates to symptom domains and functional outcomes. Understanding these relationships may provide insight into the mechanistic links between altered bodily self-awareness, emotional dysregulation, and disturbances in self-processing—features central to the psychopathology of schizophrenia. The neuroimaging component is designed to provide critical insights into the neural mechanisms underpinning interoceptive impairment. Resting-state fMRI metrics such as ALFF, ReHo, and seed-based connectivity will offer complementary perspectives on intrinsic neural activity and large-scale network organization, while diffusion tensor imaging will allow evaluation of white-matter microstructure supporting interoceptive signaling. These multimodal neuroimaging measures may help identify neural signatures associated with interoceptive deficits, contributing to the development of imaging-based biomarkers for schizophrenia.

Importantly, this study may also have clinical implications. By identifying specific brain regions and networks associated with interoceptive dysfunction, this study may help clarify the neural mechanisms underlying altered bodily self-processing in schizophrenia. Such mechanistic insights could inform the development and refinement of targeted interventions, including body-awareness-based and mindfulness-based approaches, which are thought to engage interoceptive and self-regulatory neural systems. In addition, identifying neural correlates of interoceptive deficits may help guide neuromodulation strategies by providing potential circuit-level targets for intervention and enabling more mechanistically informed and personalized treatment approaches.

Several considerations should be acknowledged. As a cross-sectional protocol, the study will not permit causal inferences regarding the directionality between interoceptive dysfunction and neural abnormalities. Although medication effects will be statistically controlled, they may still influence behavioral and neural outcomes. Antipsychotic medications may affect autonomic nervous system function, including heart rate and heart rate variability, which could potentially influence performance on interoceptive tasks such as the heartbeat counting task. Although medication dosage will be statistically controlled using chlorpromazine equivalents, residual medication-related effects cannot be completely excluded and should be considered when interpreting the findings.

Additionally, the relatively modest sample size may limit statistical power for detecting subtle neural effects or conducting reliable individual-level or subgroup analyses. Although the planned sample size is consistent with comparable multimodal neuroimaging studies, neuroimaging findings in this study should therefore be interpreted with caution. Accordingly, the primary analyses will focus on group-level comparisons and brain–behavior associations, which provide greater statistical robustness and are commonly used in neuroimaging research with similar sample sizes. Neuroimaging analyses in this protocol are primarily exploratory and will be interpreted cautiously with appropriate correction for multiple comparisons.

Furthermore, T1-weighted structural MRI will be used to assess brain morphology, diffusion tensor imaging will be used to evaluate white-matter microstructure, and resting-state functional MRI will be used to measure blood-oxygenation-level-dependent (BOLD) signal fluctuations, which represent indirect physiological indicators of neural activity rather than direct neuronal activity. In addition, concurrent cardiac and respiratory signals were not recorded during MRI scanning, which prevented the implementation of physiological noise correction methods such as RETROICOR. Finally, the neuroimaging protocol includes only resting-state fMRI rather than task-based paradigms specifically targeting interoceptive processing. Task-based paradigms may provide more direct evidence of neural responses to interoceptive signals; however, resting-state fMRI was selected to reduce task-related burden and potential performance variability in individuals with schizophrenia, who may experience difficulties maintaining attention or completing complex tasks during MRI scanning. Future studies may incorporate task-based interoceptive paradigms to further investigate neural responses to interoceptive signals.

## Glossary

ACC: Anterior cingulate cortex
AD: Axial diffusivity
AFT: Animal Fluency Test
ALFF: Amplitude of low-frequency fluctuations
BPQ: Body Perception Questionnaire
CPZ-equivalent: Chlorpromazine-equivalent antipsychotic dose
CSF: Cerebrospinal fluid
DTI: Diffusion tensor imaging
DSST: Digit Symbol Substitution Test
FA: Fractional anisotropy
FC: Functional connectivity
FDR: False discovery rate
FSL: FMRIB Software Library
FWHM: Full width at half maximum
GRETNA: Graph Theoretical Network Analysis Toolkit
GRF: Gaussian random field correction
HAM-A: Hamilton Anxiety Rating Scale
HAM-D: Hamilton Depression Rating Scale
HCT: Heartbeat Counting Task
MAIA: Multidimensional Assessment of Interoceptive Awareness
MD: Mean diffusivity
MNI: Montreal Neurological Institute standard space
MRI: Magnetic resonance imaging
PANSS: Positive and Negative Syndrome Scale
r-sfMRI/rs-fMRI: Resting-state functional magnetic resonance imaging
RD: Radial diffusivity
ReHo: Regional homogeneity
SPM: Statistical Parametric Mapping
TMT: Trail Making Test
WM: White matter.
