# Supplementary material for: Interoceptive dysfunction and its neural correlates in schizophrenia: protocol for a cross-sectional multimodal MRI study
Source: Front Psychiatry. 2026 Apr 17;17:1766350. doi: 10.3389/fpsyt.2026.1766350 (PMC13133008; doi:10.3389/fpsyt.2026.1766350)
Supplement: Supplementary file 1 [file SupplementaryFile1.docx]

# Table S1. MRI Acquisition Parameters

| **Sequence** | **Parameter** | **Value** |
| --- | --- | --- |
| Structural MRI (T1-weighted MPRAGE) | TR | 2530 ms |
| Structural MRI (T1-weighted MPRAGE) | TE | 3.65 ms |
| Structural MRI (T1-weighted MPRAGE) | Flip angle | 7° |
| Structural MRI (T1-weighted MPRAGE) | Voxel size | 1.0 × 1.0 × 1.0 mm³ |
| Structural MRI (T1-weighted MPRAGE) | Field of view (FOV) | 256 × 256 mm |
| Structural MRI (T1-weighted MPRAGE) | Slice thickness | 1 mm |
| Structural MRI (T1-weighted MPRAGE) | Number of slices | 224 |
| Resting-state fMRI | TR | 2000 ms |
| Resting-state fMRI | TE | 30 ms |
| Resting-state fMRI | Flip angle | 77° |
| Resting-state fMRI | Voxel size | 3.0 × 3.0 × 3.0 mm³ |
| Resting-state fMRI | FOV | 220 × 220 mm |
| Resting-state fMRI | Slice thickness | 3 mm |
| Resting-state fMRI | Number of slices | 50 |
| Resting-state fMRI | Band-pass filter | 0.01–0.08 Hz |
| Diffusion Tensor Imaging (DTI) | TR | 4503 ms |
| Diffusion Tensor Imaging (DTI) | TE | 80 ms |
| Diffusion Tensor Imaging (DTI) | Flip angle | 90° |
| Diffusion Tensor Imaging (DTI) | Voxel size | 2.0 × 2.0 × 2.0 mm³ |
| Diffusion Tensor Imaging (DTI) | FOV | 220 × 220 mm |
| Diffusion Tensor Imaging (DTI) | Slice thickness | 2 mm |
| Diffusion Tensor Imaging (DTI) | Number of slices | 74 |
| Diffusion Tensor Imaging (DTI) | Diffusion directions | 64 |
| Diffusion Tensor Imaging (DTI) | b-values | 0, 500, 1000, 3000 s/mm² |

**Table S2. Summary of Outcome Measures and Variables**

| **Domain** | **Measures / Instruments** | **Variables Extracted** | **Outcome Level** |
| --- | --- | --- | --- |
| Interoception (Objective) | Heartbeat Counting Task (HCT) | Accuracy score (25s, 35s, 45s) | Primary |
| Interoception (Subjective) | MAIA | 8 subscale scores | Secondary |
| Interoception (Subjective) | BPQ | Total + subscale scores | Secondary |
| Objective–Subjective Integration | HCT × MAIA × BPQ | Correlations | Secondary |
| Clinical Symptoms | PANSS | Positive/Negative/General/Total | Secondary |
| Clinical Symptoms | HAM-A / HAM-D | Total scores | Secondary |
| Cognition | TMT-A, DSST, AFT | Reaction time / Total correct | Secondary |
| Time Estimation | Time Estimation Task | Absolute & relative error | Exploratory |
| Intrinsic Neural Activity | ALFF / ReHo | Voxel-wise metrics | Exploratory |
| Functional Connectivity | Seed-based FC | Z-transformed FC matrices | Exploratory |
| Structural Connectivity | DTI (FA/MD/RD/AD) | TBSS skeleton values | Exploratory |
| Multimodal Integration | rs-fMRI + DTI + behavioral + clinical | Regression / partial correlations | Exploratory |

# Supplementary Text S1. Interoception Task Procedures

## 1. Heartbeat Counting Task (HCT)

The Heartbeat Counting Task (HCT) was administered as an objective measure of interoceptive accuracy, following the standardized protocol described by Schandry (1981).

Procedures:

• Participants were seated comfortably in a quiet room.

• Instructions emphasized focusing attention internally on heartbeat sensations.

• Participants were explicitly instructed not to check their pulse manually or use estimation strategies (e.g., counting, tapping).

• Three heartbeat perception intervals were administered: 25 s, 35 s, and 45 s, in randomized order.

• After each interval, participants verbally reported the number of heartbeats perceived.

• A 30‑second practice trial was completed prior to the test.

Outcome measure:

Interoceptive accuracy will be calculated using the standard formula:

Accuracy = 1 – (|recorded − counted| / recorded)

Higher scores reflect greater interoceptive accuracy.

## 2. Subjective Interoception Questionnaires

Participants completed two validated self-report instruments to assess subjective interoceptive awareness:

• Multidimensional Assessment of Interoceptive Awareness (MAIA):

Assesses eight dimensions of interoceptive experience, including attention regulation, emotional awareness, body listening, and trusting bodily sensations.

• Body Perception Questionnaire – Short Form (BPQ-SF):

Measures awareness of autonomic reactivity and physiological symptoms, providing both total and subscale scores.

All subscale and total scores were extracted for analysis.

## 3. Time Estimation Task

The Time Estimation Task was included as an exploratory measure of temporal perception, which is theoretically linked to interoception and internal predictive processing.

Procedures:

• Participants completed three time‑estimation intervals: 19 s, 37 s, and 49 s.

• They were instructed to estimate the duration of each interval without counting, tapping, or using motor-based strategies.

• After each interval, participants verbally provided their estimated duration.

• Three practice trials were conducted before the formal assessment.

Outcome variables:

• Absolute estimation error = |estimated − actual|

• Relative estimation error = (estimated − actual) / actual

This task is grounded in theoretical models linking interoception with temporal processing and predictive coding (Penney, 2008; Allman & Meck, 2012).


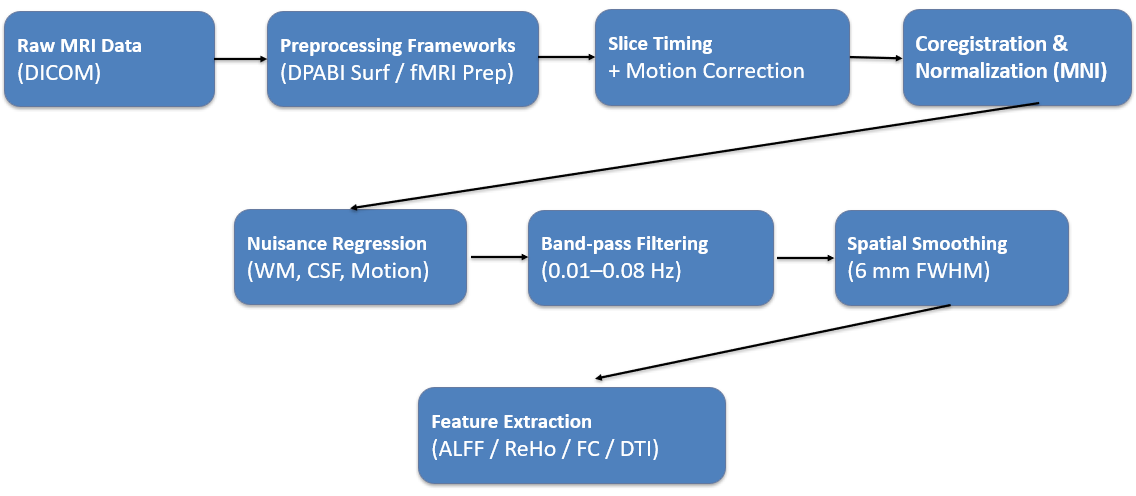


Supplementary Figure S1. MRI Preprocessing Pipeline


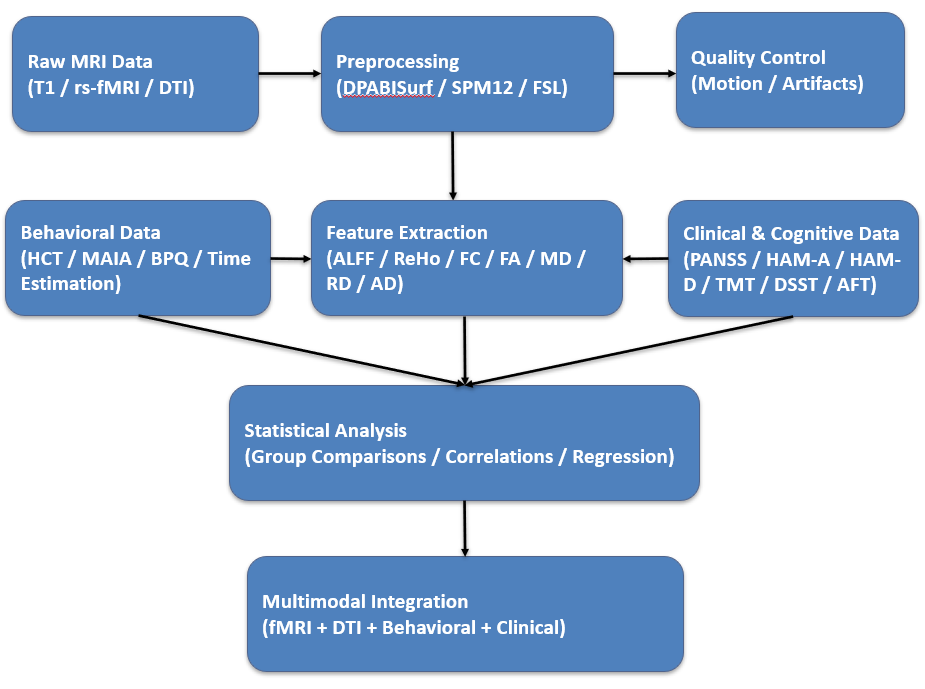


Supplementary Figure S2. Statistical Analysis and Multimodal Integration Pipeline

# Supplementary Material 1. Case Report Form (CRF)

## A. Screening & Eligibility Checklist

| Item | Value / Check |
| --- | --- |
| Age 18–65 |  |
| Right-handed |  |
| Meets DSM-5 criteria for schizophrenia (patient group) |  |
| No psychiatric disorder (control group) |  |
| No neurological disease or severe head injury |  |
| No substance or alcohol use disorder (past 6 months) |  |
| No major endocrine/cardiovascular disease affecting interoception |  |
| Not pregnant or breastfeeding |  |
| No MRI contraindications |  |

## B. Demographic Information

| Item | Value / Check |
| --- | --- |
| Participant ID |  |
| Sex |  |
| Age |  |
| Years of Education |  |
| Handedness |  |
| Height / Weight (optional) |  |

## C. Clinical Information (Patients)

| Item | Value / Check |
| --- | --- |
| Illness Duration (years) |  |
| Age of Onset |  |
| Number of Hospitalizations |  |
| Current Medication |  |
| Antipsychotic Dose (CPZ equivalents) |  |

## D. Clinical Symptom Ratings

| Item | Value / Check |
| --- | --- |
| PANSS Total Score |  |
| PANSS Positive / Negative / General Scores |  |
| HAM-A Score |  |
| HAM-D Score |  |

## E. Cognitive Tests

| Item | Value / Check |
| --- | --- |
| Trail Making Test (TMT-A) |  |
| Digit Symbol Substitution Test (DSST) |  |
| Animal Fluency Test (AFT) |  |

## F. Interoception Assessments

| Item | Value / Check |
| --- | --- |
| HCT — Heartbeat Count (25s) |  |
| HCT — Heartbeat Count (35s) |  |
| HCT — Heartbeat Count (45s) |  |
| HCT Accuracy Score |  |
| MAIA Subscale Scores |  |
| BPQ Scores |  |
| Time Estimation Task — Estimated Times (19s, 37s, 49s) |  |
| Absolute Error / Relative Error |  |

## G. MRI Quality Control

| Item | Value / Check |
| --- | --- |
| Head Motion — FD > 0.5 mm? |  |
| Artifacts (Y/N) |  |
| Final Quality Decision: Included / Excluded |  |
